# Glycolysis Inhibition Alleviates Cardiac Fibrosis After Myocardial Infarction by Suppressing Cardiac Fibroblast Activation

**DOI:** 10.3389/fcvm.2021.701745

**Published:** 2021-09-29

**Authors:** Zhi-Teng Chen, Qing-Yuan Gao, Mao-Xiong Wu, Meng Wang, Run-Lu Sun, Yuan Jiang, Qi Guo, Da-Chuan Guo, Chi-Yu Liu, Si-Xu Chen, Xiao Liu, Jing-Feng Wang, Hai-Feng Zhang, Yang-Xin Chen

**Affiliations:** ^1^Department of Cardiology, Sun Yat-Sen Memorial Hospital, Sun Yat-Sen University, Guangzhou, China; ^2^Guangzhou Key Laboratory of Molecular Mechanism and Translation in Major Cardiovascular Disease, Sun Yat-Sen Memorial Hospital, Sun Yat-Sen University, Guangzhou, China; ^3^Laboratory of Cardiac Electrophysiology and Arrhythmia in Guangdong Province, Guangzhou, China; ^4^Department of Cardiovascular Surgery, Sun Yat-Sen Memorial Hospital, Sun Yat-Sen University, Guangzhou, China

**Keywords:** heart failure, myocardial infarction, glycolysis, fibroblast activation, cardiac fibrosis

## Abstract

**Objective:** To explore the role of glycolysis in cardiac fibroblast (CF) activation and cardiac fibrosis after myocardial infarction (MI).

**Method:** *In vivo*: 2-Deoxy-D-glucose (2-DG), a glycolysis inhibitor, was injected into the abdominal cavity of the MI or sham mice every day. On the 28th day, cardiac function was measured by ultrasonic cardiography, and the hearts were harvested. Masson staining and immunofluorescence (IF) were used to evaluate the fibrosis area, and western blot was used to identify the glycolytic level. *In vitro*, we isolated the CF from the sham, MI and MI with 2-DG treatment mice, and we also activated normal CF with transforming growth factor-β1 (TGF-β1) and block glycolysis with 2-DG. We then detected the glycolytic proteins, fibrotic proteins, and the concentrations of lactate and glucose in the culture medium. At last, we further detected the fibrotic and glycolytic markers in human fibrotic and non-fibrotic heart tissues with masson staining, IF and western blot.

**Result:** More collagen and glycolytic protein expressions were observed in the MI mice hearts. The mortality increased when mice were treated with 2-DG (100 mg/kg/d) after the MI surgery (Log-rank test, *P* < 0.05). When the dosage of 2-DG declined to 50 mg/kg/d, and the treatment was started on the 4th day after MI, no statistical difference of mortality between the two groups was observed (Log-rank test, *P* = 0.98). The collagen volume fraction was smaller and the fluorescence signal of α-smooth muscle actin (α-SMA) was weaker in mice treated with 2-DG than PBS. *In vitro*, 2-DG could significantly inhibit the increased expression of both the glycolytic and fibrotic proteins in the activated CF.

**Conclusion:** Cardiac fibrosis is along with the enhancement of CF activation and glycolysis. Glycolysis inhibition can alleviate cardiac fibroblast activation and cardiac fibrosis after myocardial infarction.

## Introduction

Heart failure is a cardiovascular disease with high morbidity and mortality, which cause a great burden on society (1). Among the various etiologies, myocardial infarction (MI) is the most important one which is responsible for more than half of the cases (2). As cardiomyocyte is hard to regenerate after ischemia and hypoxia, fibrotic scar helps to maintain the integrity and function of the heart (3). However, excessive fibrosis reduces its compliance, thereby impairing its systolic and diastolic function (4). Consequently, it is a hotspot to find new strategies to restrict excessive fibrosis after MI (5).

As heart is the energy metabolism core in the body, alterations in cardiac energy metabolism contribute to several cardiovascular pathologies. Glycolysis is one of the major energy-yielding manners, which is enhanced when MI occurs (6). When the heart suffers ischemia, glycolysis can supply amounts of energy quickly, thus meeting the demands for heart contraction and blood transportation (7). Though glycolysis is well-investigated in heart failure (8, 9) and MI (10), few studies focus on the glycolysis in cardiac fibrosis after MI. Nevertheless, various fibrosis-related studies in other organs such as lung (11), liver (12), skin (13), and kidney (14) have reported that glycolysis contributes to the fibrotic process. Glycolysis contributes to fibroblast activation *via* several ways in fibrotic diseases. It not only produces several key metabolites responsible for CF activation, like glycine and triphosadenine, it also produced abundant lactate, which was important for the activity of proline hydroxylase, TGF-β1 and the hydroxylation of collagen (15, 16). Due to the specific hemodynamics of the heart, role of glycolysis in cardiac fibrosis after MI seems more complicated. It is well-acknowledged that cardiac fibroblast (CF) activation is the most important contributor to cardiac fibrosis after MI (17). Interestingly, our previous research demonstrates that enhanced glycolysis promotes cardiac fibroblast (CF) activation (18). Accordingly, it is of great significance to investigate the role of glycolysis in cardiac fibrosis after MI.

Thus, to explore the relationship between glycolysis and cardiac fibrosis after MI. We firstly detected the glycolysis-related proteins in the mouse MI model. Then, by delivering a glycolysis-specific inhibitor 2-Deoxy-D-glucose (2-DG), we demonstrated the role of glycolysis in the fibrotic process of the heart. At last, we also measured the glycolysis change in human fibrotic and non-fibrotic tissues, thus providing more clinical evidence.

## Materials and Methods

### Animals

C57BL/6J mice were used to perform a MI or sham surgery. Then 2-DG (100 mg/kg/d or 50 mg/kg/d; Sigma-Aldrich, #D8375) was delivered by intraperitoneal injection immediately after the surgery or started at the 4th-day after the surgery. Trans-thoracic echocardiography was performed on the 28th day after the surgery to evaluate the cardiac function. The animal use protocol was approved by Institutional Animal Care and Use Committee, Sun Yat-sen University. Detailed information was provided in the Supplementary Material.

### Human Heart Specimens

Human heart specimens were harvested during cardiac surgeries in Sun Yat-sen Memorial Hospital. Among them, the resected ventricular aneurysm tissues were used as fibrotic tissues while papillary muscle tissues from the diseased valve were served as non-fibrotic tissues. The study design was approved by the Ethics Committee of Sun Yat-sen Memorial Hospital.

### Masson Staining

Heart tissues from the mice or patients were collected and fixed in 4% paraformaldehyde in phosphate-buffered saline (PBS; Servicebio, #G4202) overnight after being perfused with cold normal saline. Then the hearts were processed for paraffin embedding and subsequently cut into slices. Slices then undergo dewaxing, rehydration and stained for collagen fibers with Masson's Trichrome staining Kit (Servicebio, #G1006) following the manufacturer's instructions. Fibrotic tissues and muscle tissues were segmented using ImageJ (NIH) and fibrosis was expressed as the percentage of fibrotic tissue in each section.

### Tissue Immunofluorescence

Slices from human or mice undergo dewaxing, rehydration, antigen retrieval and blocking. Then tissues were incubated with primary antibodies overnight, followed by fluorescigenic secondary antibodies incubation. At last, the nucleus was stained, and photos were taken with the fluorescence microscope. The fluorescence signal quantification was conducted with Image J.

### Cell Culture

Neonatal mouse CFs (NMCFs) and human CFs were used for the *in vitro* experiments. The NMCFs were separated from the ventricle of the neonatal 1–3-day-old mice as described previously (19), and the human CFs were isolated from human juvenile ventricle. The detailed separation method of the NMCFs was described in the Supplementary Material. The human CFs were purchased from Sciencell (#6310, the detail separation method could be found with the following link: https://www.sciencellonline.com/human-cardiac-fibroblasts-juvenile-ventricular.html).

Human CFs at the passage between 6 and 8 and NMCFs at passage 2–3 were pre-treated with 2-DG (1 mmol/L) for 1 h. Then human/mouse transforming growth factor-β1 (TGF-β1; PeproTech, 100-21; 10 ng/mL; TGF-β1; Novus Biologicals, 7666-MB; 10ng/mL) was added to induce an activated phenotype. Cells were treated for 48 h and harvested. For HK2 knockdown tests, NMCFs were treated with small interfering RNA against HK2 (si-HK2: GGACAAGCUACAGAUCAAAdTdT) or scrambled siRNA (negative control, NC: UUCUCCGAACGUGUCACGUdTdT). Cell medium was refreshed after 12 h treatment and harvested after 48 h cultivation.

Adult mouse CFs (AMCFs) were separated from the ventricle of the MI or sham mice with or without 2-DG treatment on the 28th day after the sham or MI surgery as described previously (20, 21). As inadequate cells could be harvested from only one heart, two hearts were isolated together and mixed to produce one group of cardiac cells with an enzymolysis approach. After the differential adherent method, primary AMCFs were harvested for the next experiments. The detailed separation method of the AMCFs was described in the Supplementary Material.

### Lactate and Glucose Detections

Lactate in the cell medium were detected with Lactate Assay Kit II (Sigma, # MAK065), and the glucose in the cell medium were detected with Glucose Colorimetric/Fluorometric Assay Kit (Biovision, #K606–100) according to the manufacturer's instructions. All results were normalized to the total protein concentration.

### Western Blot

Western blot was performed as previously described (18). Antibodies are listed in Supplementary Table 1. The western blot band density quantification was analyzed with Image J (National Institutes of Health, Maryland, USA).

### Cell Immunofluorescence

Cells were fixed with 4% paraformaldehyde (Servicebio, #G1101). After being permeabilized with 0.5% Triton X-100 (Sigma-Aldrich, #X100) for 20 min, cells were blocked with 5% bovine serum albumin (Sigma-Aldrich, #A1933). Afterwards, cells were incubated with the indicated the primary antibodies overnight and then the fluorescigenic secondary antibodies for 1 h at room temperature. At last, fluorescence were captured with the fluorescence microscope. Antibodies are listed in Supplementary Table 1. The fluorescence signal quantification was conducted with Image J.

### Statistical Analysis

Data were showed as mean ± SEM. Statistical analyses were performed with GraphPad Prism Software (version 8.0.1). Statistical comparison among multiple groups was carried out by one-way ANOVA followed by the *Bonferroni* test. Student's *t*-test was used to analyze differences between two groups. Kaplan-Meier method was used for the survival analysis, and the *log-rank* test was used for the statistical analyses. *P*-value < 0.05 indicated statistical significance.

## Results

### Glycolysis Was Increased in the Fibrotic Heart After MI

**Figure 1:**
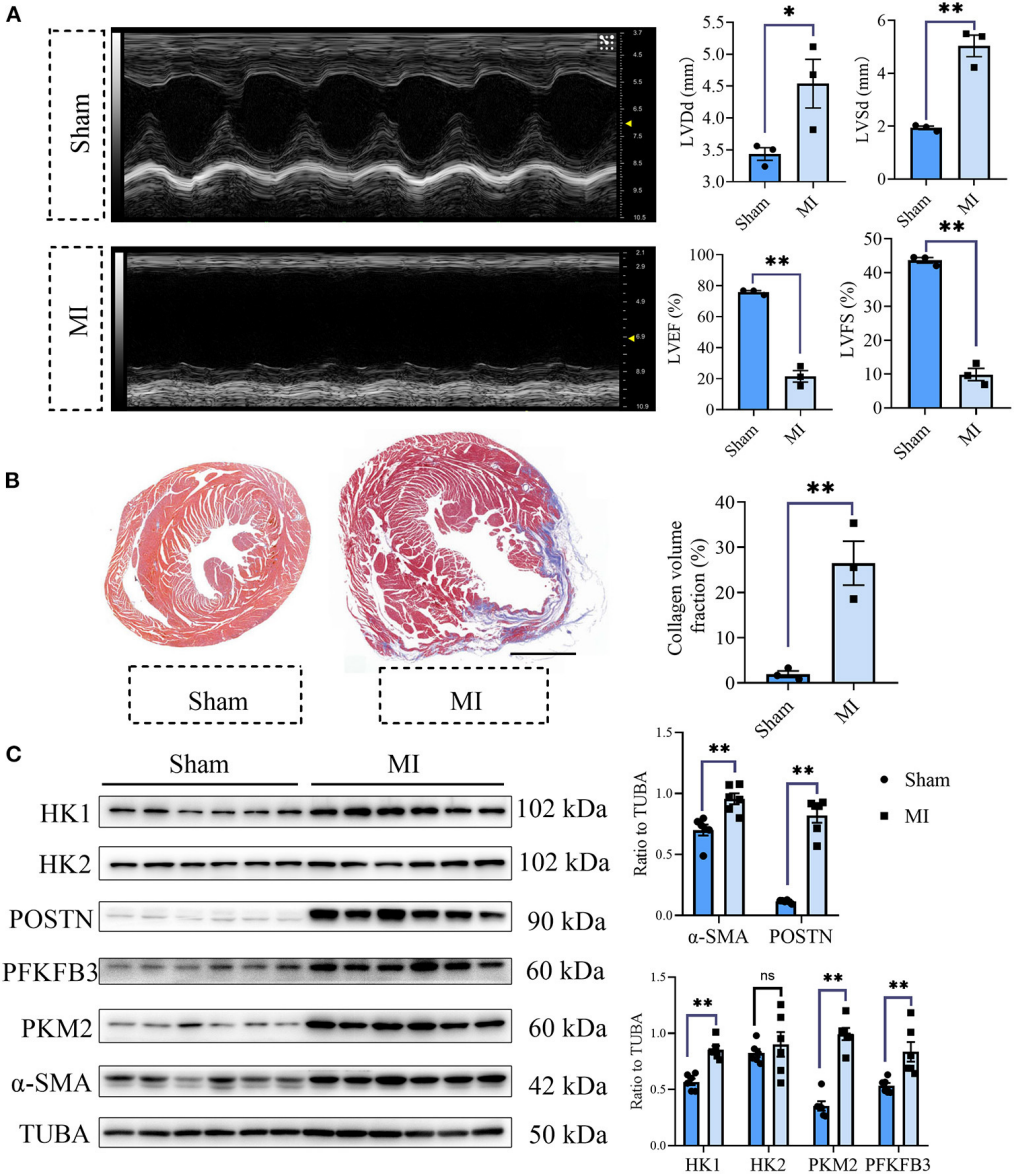
Cardiac fibrosis was accompanied by enhanced glycolysis in MI mice. **(A)** The cardiac function measured by echocardiography was shown. Representative images of the M-mode of the echocardiography results, and the related parameters (LVEF, LVFS, LVDd, LVSd) measurement were displayed, respectively. **(B)** Masson staining of the heart harvested on the 28th day after the MI or sham surgery. Representative images (left) and the statistical analysis (right) were shown. **(C)** Western blot of the expressions of glycolytic and CF activation markers between the MI and sham mice. The representative image (left) and the statistical analysis (right) were shown. LVEF, left ventricular ejection fraction; LVFS, left ventricular fractional shortening; LVDd, left ventricular end diastolic dimension; LVSd, left ventricular end systolic diameter; MI, myocardial infarction; Sham, sham operation; HK1, hexokinase 1; PFKFB3, 6-phosphofructo-2-kinase/fructose-2, 6-bisphosphatase 3; PKM2, pyruvate kinase isoform M2; POSTN, periostin; α-SMA, Alpha smooth muscle actin; *n* = 3 in **(A,B)**; *n* = 6 in **(C)**; *P < 0.05; **P < 0.01; ns, no significance. Scale bar = 1000 μm.

To explore the role of glycolysis in cardiac fibrosis, we measured the glycolytic markers in the fibrotic heart after MI. As is shown in Figure 1A, compared with the sham group, cardiac function of mice in MI group obviously decreased (sham *vs*. MI: LVEF (%): 75.84 ± 0.96 vs. 21.46 ± 3.70, LVFS (%): 43.67 ± 0.80 vs. 9.87 ± 1.83, LVDd (mm): 3.44 ± 0.10 vs. 4.54 ± 0.38, LVSd (mm): 1.94 ± 0.07 vs. 5.04 ± 0.41; *P* < 0.05). Masson staining showed that plentiful collagen was deposited in the heart after MI (collagen volume fraction (%): sham *vs*. myocardial infarction: 1.93 ± 0.71% vs. 26.49 ± 4.87%; *P* < 0.05; Figure 1B), which indicated that cardiac fibrosis occurred after MI. To further explore the change of glycolysis during this process, we harvested the heart tissue protein and detected the glycolysis-related proteins and CF activation markers. As a result, we found the increased expressions of hexokinase 1 (HK1), 6-phosphofructo-2-kinase/fructose-2, 6-bisphosphatase 3 (PFKFB3), and pyruvate kinase isoform M2 (PKM2), along with the CF activation markers (periostin, POSTN; Alpha smooth muscle actin, α-SMA) (Figure 1C). Thus, we concluded that cardiac fibrosis after MI was accompanied by enhanced glycolysis.

### Glycolysis Inhibition Alleviated Cardiac Fibrosis After MI

**Figure 2:**
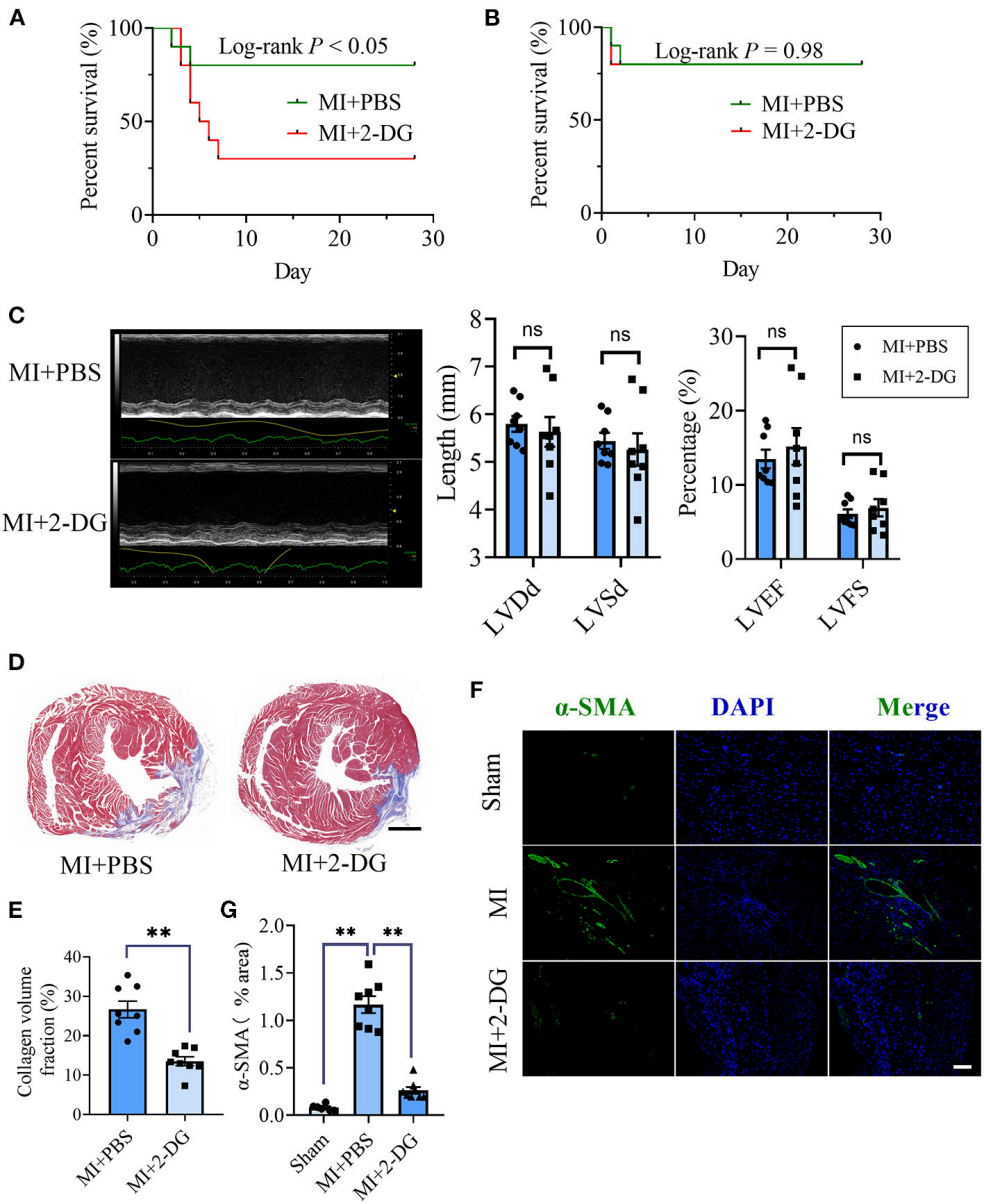
Glycolysis inhibition alleviates cardiac fibrosis after MI. **(A)** Survival analysis of the mice undergoing MI or sham surgery when 2-DG was delivered immediately after the surgery at a dosage of 100 mg/kg/d. **(B)** Survival analysis of the mice undergoing MI or sham surgery when 2-DG treatment was started on the 4th day after the surgery at a dosage of 50 mg/kg/d. **(C)** The cardiac function of the MI mice with or without 2-DG treatment at a dosage of 50 mg/kg/d started on the 4th day after the surgery was measured by echocardiography. Representative images of the M-mode of the echocardiography results (left), and the related parameters (LVEF, LVFS, LVDd, LVSd) measurement (right) were displayed, respectively. **(D,E)** The hearts of the MI mice with or without 2-DG treatment at a dosage of 50 mg/kg/d started at the 4th day after the surgery were harvested on the 28th day after the surgery. Masson staining was performed. Representative image **(D)** and the statistical analysis **(E)** were shown, respectively. **(F)** Immunofluorescence of the hearts from the MI and sham mice with or without 2-DG treatment. The green fluorescence signal represented α-SMA, and the blue fluorescence signal represented DAPI. **(G)** Fluorescence signal statistics of the green α-SMA signals. LVEF, left ventricular ejection fraction; LVFS, left ventricular fractional shortening; LVDd, left ventricular end diastolic dimension; LVSd, left ventricular end systolic diameter; MI, myocardial infarction; Sham, sham operation; *n* = 8; ns, no significance; **P < 0.01. The scale bar in **(D)** was 1000 μm. The scale bar in **(F)** was 100 μm.

Next, we aimed to figure out whether cardiac fibrosis could be reversed when glycolysis was inhibited. We delivered a glycolysis inhibitor, 2-DG (100 mg/kg/d) or an equal volume of PBS into mice in the MI group by intraperitoneal injection immediately after the surgery and maintained the injection daily. We found that the mortality increased in MI mice with 2-DG injection than that with PBS injection (Log-rank test, *P* < 0.05, Figure 2A). The animal autopsy revealed that heart rupture was the leading cause of death. Considering the possibility that glycolysis was beneficial for the early repairment of the heart, we reduced the dosage of 2-DG to 50 mg/kg/d and delayed the start time to the 4th day after the surgery. In contrast, there was no difference in mortality between the two groups (Log-rank test, *P* = 0.98, Figure 2B). Echocardiography was taken and the hearts were harvested for further experiments on the 28th day after the surgery. As is shown in Figure 2C, there was no statistical difference of cardiac function between the two groups with or without 2-DG. To further explore the role of glycolysis in cardiac fibrosis after MI, we detected collagen deposition with masson staining. Results showed that collagen volume fraction was smaller in mice treated with 2-DG than PBS (2-DG vs. PBS: 26.68 ± 2.11% vs. 13.54 ± 1.14%, *P* < 0.05; Figures 2D,E). Thus, our findings demonstrated that glycolysis inhibition could alleviate cardiac fibrosis after MI.

### Glycolysis Inhibition Could Reverse the Fibroblasts Activation *in vivo* and *in vitro*

**Figure 3:**
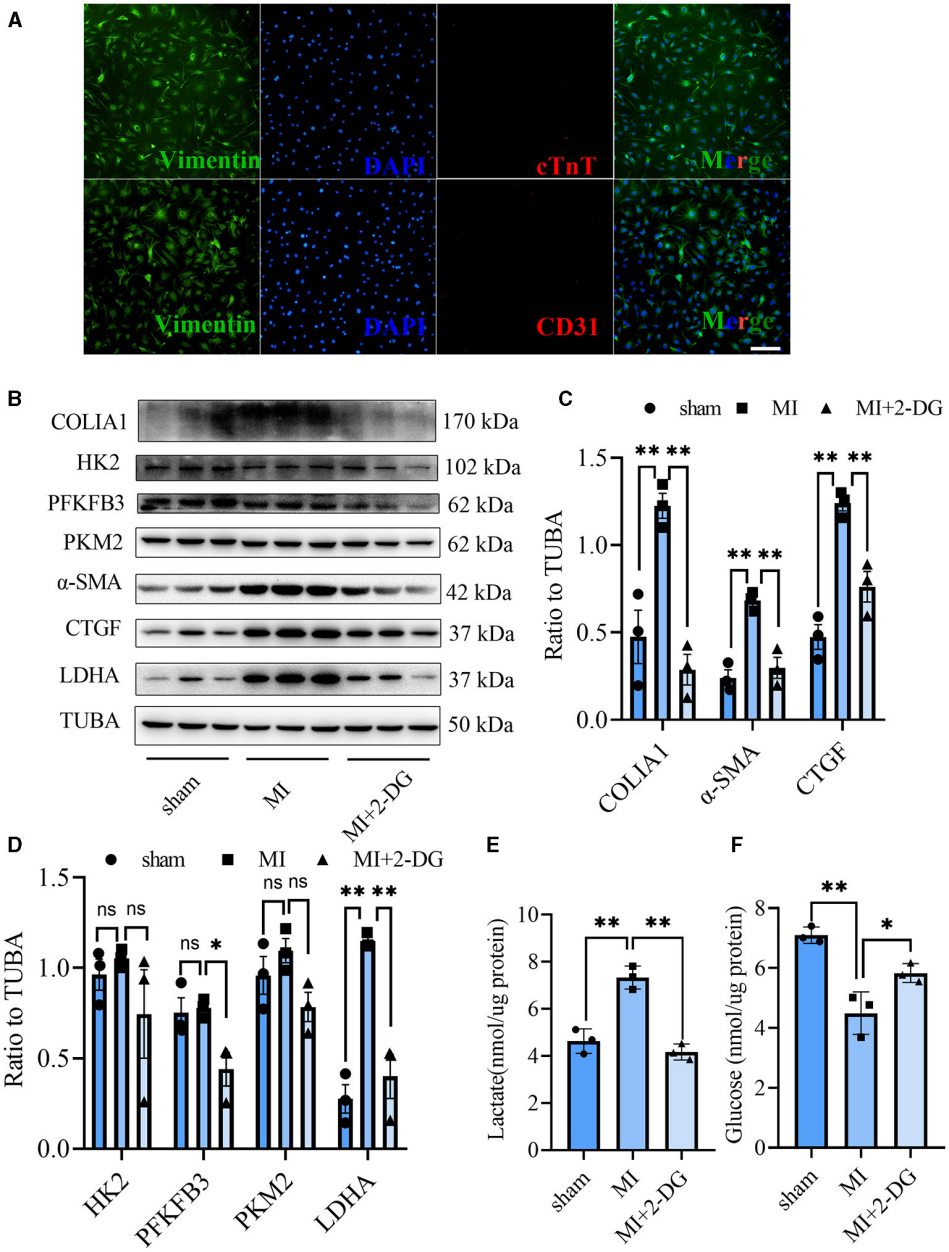
Glycolysis inhibition could reverse the fibroblasts activation *in vivo*. **(A)** Cell identification of CF isolated from the adult mice. Green represented Vimentin. Red represented cTnT or CD31. Blue represented DAPI. **(B)** Western blot of the expressions of glycolytic and CF activation markers from cells isolated from the sham, MI and MI with 2-DG treatment mice. **(C,D)** Gray value statistics of the western blot of **(B)**. **(E,F)** Lactate **(E)** and glucose measurement **(F)** in the culture medium from cells isolated from the sham, MI and MI with 2-DG treatment mice. COLIA1, type I collagen; α-SMA, alpha smooth muscle actin; CTGF, connective tissue growth factor; PFKFB3, 6-phosphofructo-2-kinase/fructose-2, 6-bisphosphatase 3; PKM2, pyruvate kinase isoform M2; HK2, hexokinase 2; TUBA, α-tubulin; LDHA, lactic dehydrogenase A; cTnT, troponin T; *n* = 3, each sample was composed of CFs from two mice hearts; *P < 0.05; **P < 0.01; ns, no significance. Scale bar = 100 μm.

**Figure 4:**
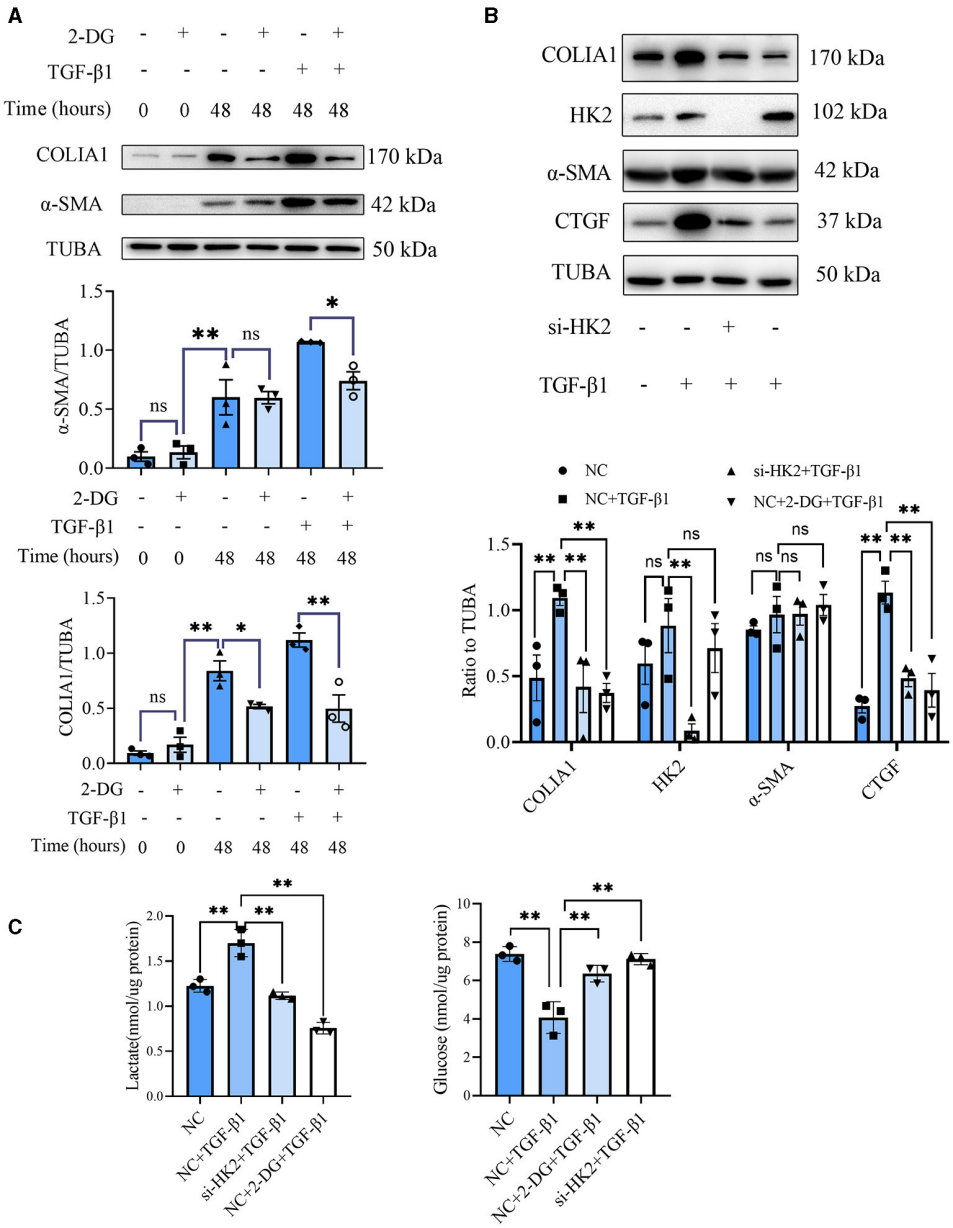
Glycolysis inhibition could reverse the fibroblasts activation *in vitro*. **(A)** Western blot of 2-DG effect on TGF-β1-induced human CF activation. **(B)** Western blot of 2-DG effect on TGF-β1-induced mouse CF activation. **(C)** Lactate and glucose measurement in the culture medium from cells. The representative image and the statistical analysis were shown, respectively. COLIA1, type I collagen; α-SMA, alpha smooth muscle actin; CTGF, connective tissue growth factor; TUBA, α-tubulin; 2-DG, 2- deoxy-D-glucose; TGF-β1, transforming growth factor-β1; NC, negative control; si-HK2, siRNA against HK2; *n* = 3, *P < 0.05; **P < 0.01; ns, no significance. Scale bar = 100 μm.

CF activation was the main force for cardiac fibrosis after MI. Thus, we then detected the markers of CF activation (α-SMA) in the heart tissues of mice in sham, MI, and MI combined 2-DG groups. As is shown in Figures 2F,G, immunofluorescent staining showed a significant reduction of α-SMA in the 2-DG treated group (MI+PBS *vs*. MI+2-DG: 1.17 ± 0.09% vs. 0.26 ± 0.03%, *P* < 0.01). To further ensure that glycolysis inhibition could alleviate the CF activation *in vivo*, we isolated the AMCFs from the MI or sham mice with or without 2-DG treatments on the 28th day after the surgery. As is shown in Figure 3A, CF isolation was successful with abundant vimentin expressions and few troponin and CD31 expressions, which were markers of CF, cardiomyocyte and endothelial cells, respectively. We then measured the expressions of the key fibrotic and glycolytic proteins. As is shown in Figure 3B, fibrotic markers such as type I collagen (COLIA1), connective tissue growth factor (CTGF) and α-SMA were significantly up-regulated in AMCFs with MI surgery. However, when the mice were treated with 2-DG after MI, the fibrotic effect exerted by MI was alleviated (Figures 3B,C). To confirm the role of glycolysis in this process, we measured the glycolytic proteins, as well as the lactate and glucose concentration in the culture medium, which were vital glycolytic indicators. Compared with the sham group, lactic dehydrogenase A (LDHA), but not PFKFB3, HK2 or PKM2, was significantly up-regulated in the MI group. Interestingly, 2-DG could reduce the expressions of both PFKFB3 and LDHA rather than HK2 and PKM2 (Figures 3B,D). More importantly, 2-DG could reverse the increase of lactate concentration and the decrease of glucose concentration in the culture medium in the MI plus 2-DG group, compared with the MI group (Figures 3E,F). Besides, we also performed an *in vitro* test to make sure whether 2-DG could inhibit CF activation directly. We used TGF-β1 to induce human cardiac fibroblast activation. In Figure 4A, 2-DG not only inhibited TGF-β1 induced CF activation, but also alleviated the CF spontaneous activation during the cultivation. We made the same conclusion with NMCFs (Figure 4B). To eliminate the off-target effects of 2-DG, we conducted the same experiments with siRNA against HK2 (si-HK2) in NMCFs. As a result, si-HK2 could also reverse the pro-fibrotic effect exerted by TGF-β1. Although si-HK2 did not influence the expression of α-SMA, it significantly decreased the expressions of COLIA1 and CTGF (Figure 4B). To better confirm the function of glycolysis in this process, we also detect the lactate and glucose concentration in the cell culture medium. As a result, the lactate concentration decreased, and the glucose concentration increased, which could be reversed with si-HK2 or 2-DG treatments (Figure 4C).

Consequently, our results demonstrated that glycolysis inhibition could reverse the fibroblast activation *in vivo* and *in vitro*.

### Glycolysis Was Increased in Human Fibrotic Heart Tissues Compared With the Non-fibrotic Heart Tissues

**Figure 5:**
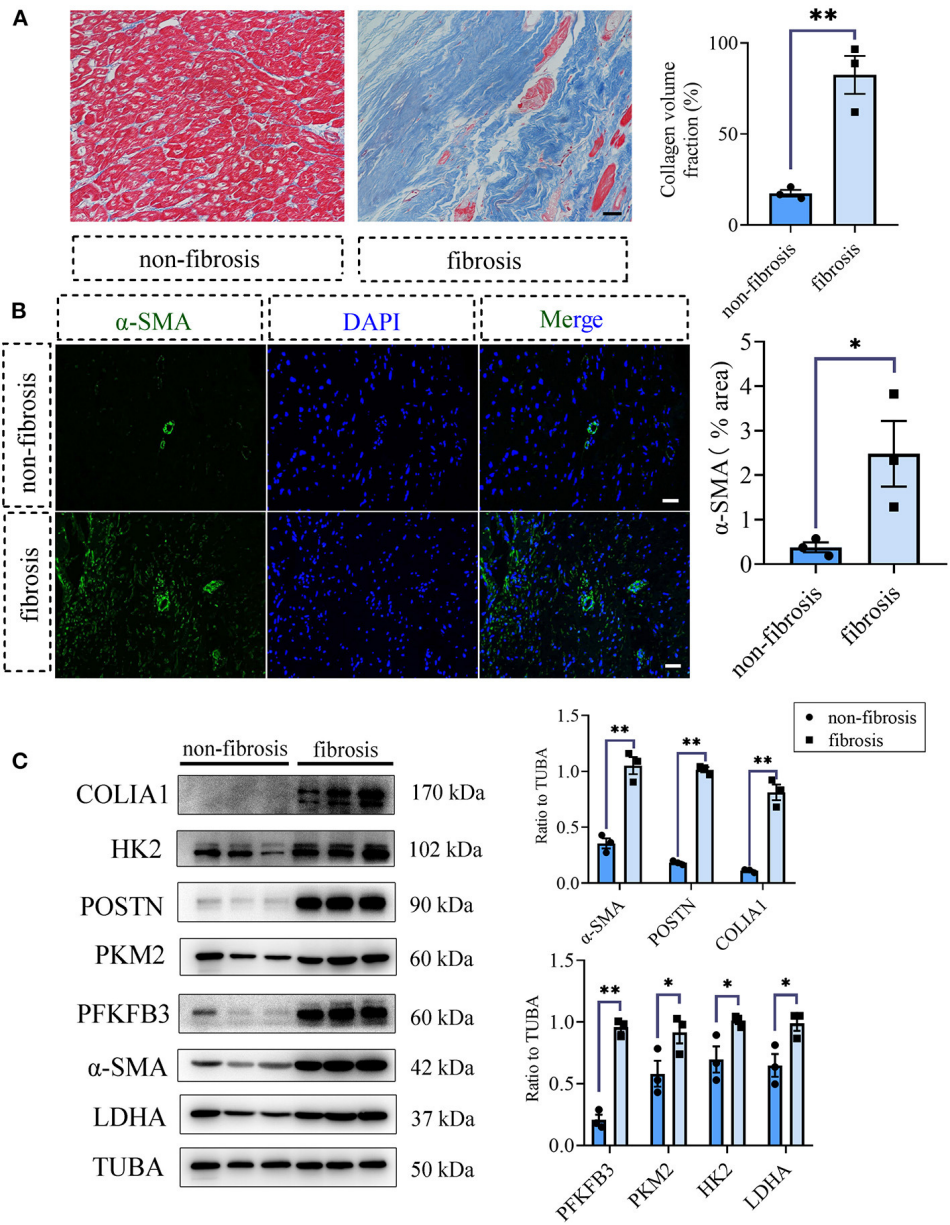
Glycolysis increased in human fibrotic heart tissues compared with the non-fibrotic heart tissues. **(A)** Masson staining of the human fibrotic and non-fibrotic heart tissues. The representative image (left) and the statistical analysis (right) were shown. **(B)** Immunofluorescence of the human fibrotic and non-fibrotic heart tissues. The green fluorescence signal represented α-SMA, and the blue fluorescence signal represented DAPI. **(C)** Western blot of the expressions of glycolytic and CF activation markers of the human fibrotic and non-fibrotic heart tissues. The representative image (left) and the statistical analysis (right) were shown, respectively. *N* = 3; *P < 0.05; **P < 0.01; ns, no significance. Scale bar = 100 μm.

To acquire the clinical evidence, we further detected the fibrotic and glycolytic markers in human fibrotic and non-fibrotic heart tissues. The clinical information of enrolled patients was shown in Supplementary Table 2. Masson staining showed that obvious collagen deposited in the human fibrotic heart tissues (collagen volume fraction (%): non-fibrotic heart tissues *vs*. fibrotic heart tissues: 17.48 ± 1.84 vs. 82.45 ± 10.45, *P* < 0.05; Figure 5A). Immunofluorescence staining showed the increased expression of α-SMA in the fibrotic hearts than the non-fibrotic hearts (α-SMA fluorescence percentage (%): non-fibrotic heart tissues *vs*. fibrotic heart tissues: 0.38 ± 0.11 vs. 2.48 ± 0.74, *P* < 0.05; Figure 5B). Consistent with the aforementioned animal experiments, fibrotic markers were increased in the human fibrotic heart tissues, which is accompanied by the increase of glycolytic markers (Figure 5C).

## Discussion

Although treatments for heart failure have made great progress, it is still a global refractory disease with high mortality and rehospitalization rate (22). Regardless of the various etiologies of heart failure, cardiac fibrosis is the common physiopathologic progress. New strategies targeting cardiac fibrosis may serve as an alternative way to improve the prognosis of heart failure.

In our study, glycolysis inhibition could alleviate the CF activation and cardiac fibrosis after MI, just as the previous reports in other fibrotic diseases (14, 23, 24). Nevertheless, in contrast to our results, Donthi et al. uncovered that the inhibition of cardiac glycolysis by overexpressing kinase-deficient PFK-2 exacerbated myocardial hypertrophy and cardiac fibrosis (25). However, when they overexpressed kinase-deficient PFK-2 with a cardiomyocyte-specific promotor, α-myosin heavy chain, glycolysis in cardiomyocyte was inhibited, which might contribute to changes in the fibrotic process indirectly (26). In another study, Hu et al. demonstrated that paroxysmal atrial fibrillation could induce glycolysis in the canine atrium, and glycolysis inhibition could completely reverse myocardial fibrosis remodeling, the results of which were consistent with our present study (27). Our findings further supported the view that glycolysis contributed to cardiac fibrosis, and glycolysis inhibition at a proper time and extent could effectively alleviate the excessive fibrosis after MI.

In our study, 2-DG delivery from the day of MI surgery at a dosage of 100 mg/kg/day increased the mortality of mice. After we lowered the dosage of 2-DG to 50 mg/kg/day and delayed the time of initial drug administration to the 4th day after the surgery, the mortality of the mice decreased, and the fibrosis area of the heart lessened. To our knowledge, fibrosis is an important event and a dynamic process after MI (28). In the first 3 days, inflammation activates along with the apoptosis of cardiomyocytes while fibroblast presents as an inflammatory phenotype by secreting plentiful inflammatory factors during this stage. From the 4th day, fibroblast tends to trans-differentiate into myofibroblasts, which drives a vast production of the extracellular matrix. In this stage, inflammation fades, and the scar arises (28). During the process of fibrosis development after MI, modest fibrosis will help the heart to complete the repairment of the heart while inadequate fibrosis may result in the increased risk of heart rupture due to the insufficient repairment. On the contrary, excessive fibrosis contributes to the stiffness of the heart, with resultant impaired systolic and diastolic functions. In our experiments, sufficient 2-DG delivery might block both the cardiomyocyte survival and the fibroblast activation, which did harm to the heart repair, thus accelerating the rupture of the heart. When the dosage of 2-DG was decreased and the delivery time was delayed, the side effect alleviates, and 2-DG can effectively inhibit the cardiac fibroblast activation, thus contributing to the improvement of the cardiac fibrosis. Anyway, the side effect of 2-DG on the cardiomyocyte exist, which may explain the little improvement in the cardiac function. Thus, cell-specific reagent invention was in need in the future studies. Searching the downstream of the 2-DG might be another alternative way to reduce the side effect of 2-DG. More works need to be done in the future to promote the application of glycolysis inhibitors in the cardiac fibrosis treatment.

In our study, we did not explore the further mechanism of the anti-fibrotic effect of 2-DG. 2-DG might contribute to CF activation in several ways. As several critical metabolites in glycolysis were responsible for CF activation, like glycine and triphosadenine (15), 2-DG might inhibit CF activation by reducing the production of these pro-fibrotic intermediate metabolites. Besides, glycolysis also produced abundant lactate, which was essential for the activity of proline hydroxylase, TGF-β1, and the hydroxylation of collagen (16). Interestingly, 2-DG could cut down the secretion of lactate significantly. As proline, collagen and TGF-β1 were important molecular for CF activation, 2-DG might exert the anti-fibrotic effects by restricting the production of lactate. What's more, glycolysis could also enhance the expression of the fibrotic proteins by epigenetic modification, which could be another intervention target for the anti-fibrotic effect of 2-DG (29). Last but not the least, hypoxia was an important change after MI, and hypoxia was a key inducer of glycolysis. In the kidney fibrosis investigation, it was reported that TEPP-46-induced PKM2 tetramer formation and pyruvate kinase activity resulted in the suppression of HIF-1α and lactate accumulation, thus contributing to kidney fibrosis (30). In another study of diabetic kidney disease, sodium-glucose cotransporter 2 inhibition could suppress HIF-1α-mediated metabolic switch from lipid oxidation to glycolysis and exert a kidney protective effect (31). It was fascinating that hypoxic signaling post MI (such as HIF1α activation) might drive this presumed glycolytic shift. More works are needed in the future study to illustrate the inner mechanism.

Some other limitations should be acknowledged in our study. Firstly, we delivered 2-DG by intraperitoneal injection, which may exert a systemic effect. Though we have demonstrated the role of glycolysis on CF activation *in vivo* and *in vitro*, we cannot eliminate the possible confounding contribution by other cardiac cells. Secondly, we explored the role of glycolysis in cardiac fibrosis and CF activation after MI. We did not further explore the role of tricarboxylic acid cycle and oxidative phosphorylation. Thirdly, as human tissues were hard to acquire, especially normal heart tissues and tissues from remote sections of the human MI heart, we had to use papillary muscles tissues as candidates of non-fibrotic heart tissues.

In conclusion, our study demonstrates that glycolysis inhibition can alleviate CF activation and cardiac fibrosis after MI. Glycolysis may be a new target for the treatment of cardiac fibrosis.

## Data Availability Statement

The raw data supporting the conclusions of this article will be made available by the authors, without undue reservation.

## Ethics Statement

The studies involving human participants were reviewed and approved by the Ethics Committee of Sun Yat-sen Memorial Hospital. The patients/participants provided their written informed consent to participate in this study. The animal study was reviewed and approved by Institutional Animal Care and Use Committee, Sun Yat-sen University.

## Author Contributions

Z-TC, Q-YG, and M-XW were responsible for most of the experiments. MW provided the human heart specimens. YJ, R-LS, and D-CG provided the experimental assistances. QG, C-YL, XL, and S-XC provided writing assistances. J-FW, H-FZ, and Y-XC designed and directed the study. All authors have read and approved the final submitted manuscript.

## Funding

This work was supported by grants from the National Natural Science Foundation of China (No. 81870170, 81970200, and 82100369), Guangdong Basic and Applied Basic Research Foundation (2020A151501886, 2019A1515110129), the Yat-sen Start-up Foundation (No. YXQH202014), the Science and Technology Program of Guangzhou City of China (201803040010), the Guangzhou Regenerative Medicine and Health Guangdong Laboratory (No. 2019GZR110406004), and the Guangzhou Key Laboratory of Molecular Mechanism and Translation in Major Cardiovascular Disease (No. 202102010007).

## Conflict of Interest

The authors declare that the research was conducted in the absence of any commercial or financial relationships that could be construed as a potential conflict of interest.

## Publisher's Note

All claims expressed in this article are solely those of the authors and do not necessarily represent those of their affiliated organizations, or those of the publisher, the editors and the reviewers. Any product that may be evaluated in this article, or claim that may be made by its manufacturer, is not guaranteed or endorsed by the publisher.
