# Supplementary material for: Glycolysis Inhibition Alleviates Cardiac Fibrosis After Myocardial Infarction by Suppressing Cardiac Fibroblast Activation
Source: Front Cardiovasc Med. 2021 Sep 29;8:701745. doi: 10.3389/fcvm.2021.701745 (PMC8511672; doi:10.3389/fcvm.2021.701745)
Supplement: Supplementary file 1 [file Data_Sheet_1.docx]

**Materials and Methods**

**Animals**

C57BL/6J mice were purchased from and maintained in Laboratory Animal Center, Sun Yat-sen University. The animal use protocol was approved by Institutional Animal Care and Use Committee, Sun Yat-sen University. Male mice underwent permanent ligation of the left anterior descending (LAD) artery or sham surgery at eight weeks old. Briefly, mice were anesthetized with 50mg/kg pentobarbital. With the help of a breathing machine, the fourth inter costal space over the left chest was exposed, the LAD artery of the MI group was ligated by a 7 sterile silk suture while that of the sham group was not. Subsequently, the chest and the overlying skin were closed, allowing the animals to recover under aseptic precautions and a warm environment. Then 2-DG (100mg/kg/d or 50mg/kg/d; Sigma-Aldrich, #D8375) was delivered by intraperitoneal injection immediately after the surgery or started on the 4^th^-day after the surgery. Trans-thoracic echocardiography was performed on the mice with Vevo 2100 (VisualSonics) on the 28^th^ day after the surgery. Images at M-mode short axis at mid papillary muscle level were taken and served for the cardiac function parameter measurements (left ventricular ejection fraction, LVEF; left ventricular fractional shortening, LVFS; left ventricular end-diastolic dimension, LVDd; left ventricular end systolic diameter, LVSd). At last, hearts were harvested after being perfused with cold normal saline for the following western blot and slices staining.

**Primary cell culture**

Neonatal mouse CFs (NMCFs) were separated from the ventricle of the neonatal 1-3-day-old mice. Briefly, ventricles were harvested from the neonatal mouse. After being incubated with 0.1% trypsin (Gibco, #25300120) overnight, the heart tissues were digested in 0.08% type 2 collagenase (Gibco, #17101015) at a temperature of 37℃ for three times. Then cells were acquired and seeded into a culture dish in an incubator containing 5% CO_2_. After differential adhesion for 30 minutes twice, NMCFs were prepared for the following experiments.

Adult mouse CFs (AMCFs) were isolated from the ventricle of the MI or sham mice with or without 2-DG treatment. As one heart produced inadequate cells, two hearts were isolated together and mixed to produce one group of cardiac cells. Briefly, freshly beating hearts were dissected and placed in the cold Krebs-Henseleit buffer (Sigma, #K3753). After incubating with collagenase digestion cocktail (containing DNase I, HEPES and collagenase) (Dnase I, BioRad, #7326828; HEPES, Corning, # 25–060-Ci; Collagenase blend, Gibco, #17101015) and 1x RBC lysis buffer (Miltenyi, #130–094-183), the solution was pipetted up and down to reach a single cell suspension and the supernatant was collected. Cardiac cells were collected by centrifugation at 1200 rpm for 5 min. The pellet was resuspended and plated in 25cm^2^ flask about 4 hours to harvest AMCFs. After this step, refresh the medium. CFs were prepared for the following experiments.

**Table 1 Antibodies used in western blot or cell immunofluorescence**

| Name | Manufacturer | Product number | Dilution Ratio |
| --- | --- | --- | --- |
| Anti-α-SMA | Abcam | ab5694 | 1:1000 |
| Anti-TUBA | Abcam | ab18251 | 1:1000 |
| Anti-CTGF | R&D | MAB91901 | 1:1000 |
| Anti-POSTN | Abcam | ab14041 | 1:1000 |
| Anti-PKM2 | CST | 4053 | 1:1000 |
| Anti-PFKFB3 | Abcam | ab181861 | 1:1000 |
| Anti-HK2 | CST | 2867 | 1:1000 |
| Anti-COLIA1  Anti-HK1  Anti-LDHA | Abcam  CST  CST | ab138492  2024  3582 | 1:1000  1:1000  1:1000 |
| cTnT | abcam | ab209813 | 1:100 |
| CD31 | abcam | ab222783 | 1:100 |
| Vimentin | cst | D21H3 | 1:100 |

COLIA1, type I collagen; α-SMA, alpha smooth muscle actin; CTGF, connective tissue growth factor; PFKFB3, 6-phosphofructo-2-kinase/fructose-2, 6-bisphosphatase 3; PKM2, pyruvate kinase isoform M2; HK2, hexokinase 2; TUBA, α-tubulin; LDHA, lactic dehydrogenase A; POSTN, periostin; HK1, hexokinase 1; cTnT, troponin T.

**Table2. Baseline clinical information of the enrolled patients**

| Clinical information | Patients without cardiac fibrosis | Patients with cardiac fibrosis |
| --- | --- | --- |
| Age | 61.3 ± 9.0 | 54.7 ± 5.7 |
| Gender (%) | 33.3 | 100 |
| Smoking (%) | 0 | 66.6 |
| Hypertension (%) | 0 | 66.6 |
| Diabetes (%) | 0 | 33.3 |
| BMI (kg/m^2^) | 22.4 ± 2.3 | 23.6 ± 2.8 |
| Triglyceride (mmol/L) | 1.9 ± 1.0 | 1.5 ± 0.3 |
| Total cholesterol (mmol/L) | 6.5 ± 0.3 | 3.7 ± 1.0 |
| LDL-c (mmol/L) | 4.3 ± 0.4 | 2.3 ± 0.8 |
| HDL-c (mmol/L) | 1.2 ± 0.1 | 1.0 ± 0.1 |
| Creatinine (µmol/L) | 91 ± 22.5 | 113.3 ± 8.0 |
| Ejection fraction (%) | 57.0 ± 6.1 | 39.7 ± 15.8 |
| Left atrium (mm) | 47.3 ± 6.7 | 42.3 ± 5.0 |
| Right atrium (mm) | 42.0 ± 6.6 | 41.0 ± 5.3 |
| LVDd (mm) | 52.3 ± 14.0 | 65.0 ± 4.4 |
| RVDd (mm) | 27.0 ± 12.5 | 31.7 ± 11.0 |

Annotation: n=3, data are shown as the mean ± SEM of the information from three patients.
